# Validity and Reliability of Farsi Version of Youth Sport Environment Questionnaire

**DOI:** 10.1155/2015/985283

**Published:** 2015-08-12

**Authors:** Mohammad Ali Eshghi, Ramin Kordi, Amir Hossein Memari, Ahmad Ghaziasgar, Mohammad-Ali Mansournia, Seyed Hojjat Zamani Sani

**Affiliations:** ^1^Exercise Physiology Research Center, Baqiyatallah University of Medical Sciences, Tehran, Iran; ^2^Sports Medicine Research Center, Tehran University of Medical Sciences, P.O. Box 14395-578, Tehran, Iran; ^3^Department of Epidemiology and Biostatistics, School of Public Health, Tehran University of Medical Sciences, Tehran, Iran; ^4^Faculty of Physical Education and Sport Sciences, Shahid Beheshti University, Tehran, Iran

## Abstract

The Youth Sport Environment Questionnaire (YSEQ) had been developed from Group Environment Questionnaire, a well-known measure of team cohesion. The aim of this study was to adapt and examine the reliability and validity of the Farsi version of the YSEQ. This version was completed by 455 athletes aged 13–17 years. Results of confirmatory factor analysis indicated that two-factor solution showed a good fit to the data. The results also revealed that the Farsi YSEQ showed high internal consistency, test-retest reliability, and good concurrent validity. This study indicated that the Farsi version of the YSEQ is a valid and reliable measure to assess team cohesion in sport setting.

## 1. Introduction

Team cohesion is one of the key concepts within sport teams that has received substantial research attention over the last three decades [1, 2]. Team cohesion is defined as a dynamic process addressing the propensity for a team to bond together and remain integrated in chasing their purposes [3]. Team cohesion is directly associated with other main sport measures such as collective efficacy, performance, and achievement. Carron et al. [4] presented the first conceptual model of team cohesion in Group Environment Questionnaire (GEQ) including two categories: team integration and individual attraction to the team. Each category was divided into two subcategories known as social cohesion and task cohesion. Social cohesion was related to the extent team athletes adhere together to show appropriate social relationships, while task cohesion is indicative of joint effort among team members in order to reach specific team goals [5, 6].

The usefulness of the GEQ has been frequently examined. Researchers have assessed factor structure and construct validity of the GEQ across different cultures [7–9]. Results have questioned the validity of the GEQ for young individuals as whether the GEQ is generalizable to young athletes' population [10]. Seeking to answer the question, researchers examined the appropriateness of the GEQ items for youth athletes and revised questions to outfit if necessary. Accordingly, Eys et al. [11] developed a cohesion questionnaire for exclusively investigating young athletes (≤17) named the Youth Sport Environment Questionnaire (YESQ). Eys et al. [11] and other researchers believed that original conceptualization of GEQ might not be relevant to the young population [12, 13]. Since the developmental variables play a critical role in perceptions of youth about their peer interactions and relationships, team experiences in young athletes become increasingly diverse, complex, and integrated through developmental process [14]. Consequently and of relevance to the current study, Eys et al. [11] believed that young athletes may not perceive group unity from two aspects of team integration and individual attraction to the team but instead identify an unidimensional model of team cohesion. Furthermore, Eys et al. [11] emphasized item wording to increase readability and decrease response bias in younger individuals. Eys et al. [15] also reported that certain individual characteristics (e.g., age) may influence both the participant's ability to interpret mixed or negatively worded questions and the internal consistency values of the GEQ subscales.

In sum, the literature reviewed above highlights the existing question of whether the YSEQ is effective as a measure of cohesion across age, culture, and youth sport teams [2]. However there is a lack of research on team cohesion in young athletes particularly outside of English-spoken countries [7]. This gap stems from lack of a valid, reliable, and relevant measure of the cohesion construct for youth sport groups. Therefore, the purpose of the current study was to continue the validation procedure of a sound measure of cohesion in order to assess the perception of cohesion in members of youth (13–17 years of age) sport groups using the Farsi version of the YSEQ. To meet this goal a sample of Iranian young athletes was recruited for the study.

## 2. Methods

### 2.1. Participants

The studied sample included 455 healthy youths (253 male) with an average age of 15.1 years (SD = 1.8). A convenience sample of athletes was recruited from local sports leagues. Coaches were initially contacted and upon their approval researchers explained the study for all athletes. Volunteered male or female athletes were assigned to the study if they were 13–17 years old and participated on competitive levels in karate, basketball, volleyball, and soccer.

### 2.2. Measures

In order to collect descriptive information, participants were asked to fill a questionnaire that consisted of demographic information such as gender, occupation, and age as well as exercise information including type of sport, number of participations per week, and duration of training sessions.

### 2.3. The Youth Sport Environment Questionnaire

The YSEQ is a modified form (18-item) of the validated version of the GEQ. The YSEQ also measures the team cohesion of the participants on a 9-point Likert scale ranging from 1 (*strongly disagree*) to 9 (*strongly agree*) (see Supplementary Material available online at http://dx.doi.org/10.1155/2015/985283). Scoring in the YSEQ is the same as the GEQ, but the items have just been categorized into two dimensions of* social cohesion* and* task cohesion*.

### 2.4. Sport Competition Anxiety Test

To test the concurrent validity, the Sport Competition Anxiety Test (SCAT) was used to measure competitive trait-anxiety through 15 questions [16]. The questions begin with “Before I compete I feel…” and athletes were asked to respond to each statement by checking a box corresponding to the frequency (hardly ever, sometimes, or often). The test score ranges from 10 (low competitive trait-anxiety) to 30 (high competitive trait-anxiety).

### 2.5. Shortened Marlowe-Crowne Social Desirability Scale (SMCSDS)

For assessing social desirability bias in participants' responses, a 13-item short form of the SMCSDS was applied [17]. Social desirability bias suggests that respondents tend to show socially favorable and acceptable image of themselves to others when answering self-evaluative questions. The correlation between the SMCSDS and the YSEQ was obtained to reveal if answers were affected by social desirability bias.

### 2.6. Procedure

The Farsi version of the YSEQ was translated from the original language (English) to Farsi by two different independent bilingual translators whose first languages were Farsi [18]. One of the translators was a psychologist experienced in translating sport psychology questionnaires; she was aware of the purpose and the concepts applied in the questionnaire. The other translator had no psychological background and was uninformed about the purpose of study. Both translators discussed differences between translations and resolved inconsistencies. Ultimately they made a final version of translation upon which both agreed. The back translation of the Farsi version to English was made by one bilingual translator whose mother tongue was English and was experienced in the field of psychology. Afterward, a multidisciplinary expert committee evaluated and reviewed the final Farsi translation and the back translation. The committee consisted of the translators, a sport researcher, a psychologist, and a statistician. The expert committee reviewed and evaluated all the translations and compared them to the original English version and concluded that translations were idiomatically, semantically, and culturally equivalent. They resolved discrepancies and reached to a consensus to form the prefinal version of the Farsi version of the YSEQ. After finalizing the last draft, it was pretested in a sample of 25 young athletes in order to assess the clarity of the statements and the questionnaire. Based on the feedback we received, changes were made to the semantics of some statements in order to ensure understandability of the questionnaire for the Iranian population.

The Institutional Review Board of the Tehran University of Medical Sciences approved ethical aspect of this research. The participants after completing an informed consent received three questionnaires including YSEQ, SCAT, and SMCSDS and a demographic information form. Participants were briefed that there is no wrong or right answer and they should respond based on their experience.

### 2.7. Statistical Analysis

We conducted a confirmatory factor analysis (CFA) on the 18-item version of the questionnaire examining the final two-factor model parallel to one-factor model suggested by Eys et al. [11] study. A maximum likelihood method of estimation by AMOS 17.0 was used for confirmatory factor analysis. The concurrent validity was assessed to verify if the Farsi version of the YSEQ measures is in line with other psychological measures in the sport context. Thus we correlated each subscale of the YSEQ with the SCAT scores using Spearman's rank correlation coefficient. Furthermore, to assess the social desirability bias in participants we used Pearson's product-moment correlation between the YSEQ subscales and the SMCSDS. Internal consistency was assessed using Cronbach's coefficient alpha. Additionally “Cronbach's alpha if item deleted” was reported. In the “item total correlation,” high correlation between item and total score for the subscale represents that the item is a powerful item. The YSEQ was readministered to 60 participants after an interval of four weeks. The test-retest reliability was then evaluated using intraclass correlation coefficient. IBM SPSS software version 17 was used for analysis and an alpha value of 0.05 was considered as the statistical significance level.

## 3. Results

### 3.1. Confirmatory Factor Analysis

Descriptive statistics and standardized factor loadings of all items are presented in Table 1. CFA was conducted to test goodness of fit for each one-factor and two-factor model of the Farsi YSEQ. Goodness of fit summary for one- and two-factor models is shown in Table 2 and the correlation matrix on which these CFAs were based is presented in Table 3.* The fit indices for* one-factor structure* showed a poor* fit of the model: *χ* ^2^ = 543.356 (*p* < 0.001), DF = 170, *χ* ^2^/df = 3.102, TLI = 0.68, NFI = 0.75, CFI = 0.81, IFI = 0.78, and RMSEA = 0.084 with 90% CI = 0.075–0.093. However the two-factor structure (social and task cohesion) indicated adequate fit of the model to the data, with *χ* ^2^ = 313.425 (*p* < 0.001), DF = 169, *χ* ^2^/df = 1.854, TLI = 0.93, NFI = 0.89, CFI = 0.92, IFI = 0.94, and RMSEA = 0.051 with 90% CI = 0.043–0.062. The significant chi-square test was highly likely with large sample sizes. Furthermore, examination of the standardized factor loadings suggested that two items had a low value (i.e., 6 and 12; spurious negative items). These items had been added to detect invalidating response sets. Therefore those items were not included in the final model.

### 3.2. Reliability

The results showed a high internal consistency (*α* = 0.93) of the YSEQ for the total items. The item total correlation for social subscale ranged from 0.67 to 0.78 and item deleted alpha coefficients varied from 0.83 to 0.87 while overall coefficient alpha for this subscale was 0.87. The item total correlation for task subscale was between 0.61 and 0.73 and item deleted alpha coefficients were ranging from 0.83 to 0.85 with internal consistency coefficient of 0.89. All of the items had adequate correlation in relation to each subscale. Analysis also showed a high test-retest reliability of the Farsi version of the YSEQ after one month; intraclass correlation coefficient for the social and task scores was 0.89 and 0.92, *p* < 0.001.

### 3.3. Concurrent Validity

When examining concurrent validity, the Farsi version of the YSEQ was significantly correlated with the SCAT scores. High negative correlations were found between SCAT and the YSEQ subscales for task scores (*r* = −0.59, *p* < 0.001) and social scores (*r* = −0.48, *p* < 0.001). Furthermore, correlation between each YSEQ subscale and the SMCSDS was very low, ranging from 0.015 to 0.18 and these results confirmed the participants' answers were not influenced by social desirability.

## 4. Discussion

The YSEQ is a self-report instrument that has been recently used for measurement of team cohesion [11]. This study aimed to evaluate validity and reliability of the Farsi version of the YSEQ. From proposed models, two-factor structure (task and social cohesion) of the scale shows better fit indices compared with one-factor structure.

### 4.1. Validity

The current study findings on cohesion are comparable to those in previous reports indicating that younger athletes showed a specific perception on team cohesion [2]. The YSEQ was initially based on a strong theoretical foundation provided by Carron and colleagues [4] that is supported by over two decades of research; but in the initial version of the YSEQ the youth's perception on constructs of the group integration and attractions to the group were not separated. However, task and social differences were still clearly distinguished both conceptually and statistically. This can be due to the level of complexity in which youths athletes conduct their interactions and relationships with others in a sport team [19]. Furthermore there are questions about the overall utility of examining individual perceptions related to a group in the cohesion research. The distinction between task and social issues in the YSEQ is in line with a number of previous group dynamics studies that have indicated these issues as two primary orientations for most populations [20–22]. Examining cohesion in other disciplines (such as work groups) also supported the differences between task cohesion and social cohesion [23]. Similar to the original work, and in addition to the task and social items, two false negative questions have been added to address concerns about the response agreement and negative versus positive wording of items. Addressing the readability of statements, in line with the original study, current study provided further support for the appropriateness of the language for a youth sample with a different culture.

Examining the concurrent validity of the Farsi version of the YSEQ, current study indicated a strong negative correlation between subscales of the Farsi version of the YSEQ and the SCAT scores. This result is in line with other studies in which authors argued that cohesion is influenced negatively by the anxiety and distress of athletes related to their competitions [24]. Examining the scores of the MCSDS in relation to the subscales of the YSEQ indicated a very weak correlation. This indicates that the YSEQ questions did not affect athletes to answer questions with a social desirability tendency. Thus their responses could be assumed as indicators of their real intention. This suggests that the YSEQ might not bias participants to overestimate their team abilities [2].

### 4.2. Reliability

Current study results have also reported a high internal consistency for the total score and each factor of the YSEQ, confirming the stability of the scale. We also showed that total item correlations for subscales were satisfactory and each subscale was internally stable. Furthermore, test-retest reliability revealed a high stability for the Farsi version of the YSEQ after four weeks.

### 4.3. Strengths and Limitations

The strength of our study was to study athletes from a wide range of sports [25]. This has led to a high validity, robust factor structure, and internal reliability of both task and social cohesion subscales. However, the validation of YSEQ will be continuing and future research should investigate the predictive function of the YSEQ for theory-based concepts related to a sport team. A limitation of this study, which might be addressed by future research studies, is about the concurrent validity where we used only one scale instead of multiple designs. It would be more advantageous to correlate the YSEQ with other sport team measures scales in Farsi (e.g., role clarity, collective efficacy) for assessing concurrent and discriminant validity [26]. However there are several individual variables associated with cohesion particularly variables as competitive anxiety. Anxiety as an affective-cognitive variable is associated with task cohesion and athletes in cohesive teams will experience less anxiety. Based on previous data, it could be assumed that competitive anxiety is a fitting outcome variable for team cohesion. Thus our findings on association between cohesion and anxiety could improve the predictive value for the YSEQ.

## 5. Conclusion

In conclusion, findings of the current study indicated that the Farsi version of the YSEQ is a sound measure to assess perceptions of cohesion among youth athletes (13–17 years of age) from a range of sport groups. From a practical standpoint, current findings imply that psychologists can use the Farsi version of the YSEQ to assess overall task or social group cohesion in their young athletes.

## Supplementary Material

Supplementary Material: Example items from the Youth Sport Environment Questionnaire.

## Figures and Tables

**Table 1:** Descriptive statistics and standardized factor loadings for confirmatory factor analysis (*N* = 455).

Factor	Item	Loading	Range	Mean	SD
Social cohesion	2	0.69	2–9	6.45	2.09
	4	0.68	1–9	6.21	2.13
	7	0.67	2–9	5.98	1.98
	9	0.73	1–9	6.87	2.35
	11	0.71	1–9	6.98	2.47
	13	0.84	1–9	5.57	1.99
	15	0.86	1–9	5.37	2.15
	17	0.81	1–9	6.61	2.24

Task cohesion	1	0.59	2–9	7.05	2.16
	3	0.67	1–9	6.55	2.24
	5	0.76	1–9	6.84	2.18
	8	0.63	1–9	5.98	1.95
	10	0.84	2–9	6.37	2.04
	14	0.77	2–9	7.09	2.07
	16	0.82	1–9	6.79	1.89
	18	0.74	1–9	6.13	1.97

**Table 2:** Correlation matrix of the 16 items used in the confirmatory factor analyses.

Item	2	4	7	9	11	13	15	17	1	3	5	8	10	14	16	18
Social 2	—	0.56	0.48	0.51	0.44	0.57	0.44	0.47	0.18	0.32	0.31	0.28	0.24	0.30	0.27	0.23
Social 4		—	0.49	0.58	0.53	0.52	0.49	0.51	0.21	0.33	0.26	0.28	0.27	0.30	0.32	0.19
Social 7			—	0.54	0.48	0.51	0.43	0.47	0.22	0.23	0.27	0.26	0.24	0.33	0.31	0.22
Social 9				—	0.47	0.43	0.46	0.38	0.15	0.21	0.31	0.16	0.18	0.24	0.19	0.20
Social 11					—	0.38	0.41	0.43	0.16	0.23	0.24	0.19	0.24	0.27	0.26	0.17
Social 13						—	0.53	0.49	0.28	0.14	0.19	0.23	0.22	0.31	0.25	0.15
Social 15							—	0.55	0.17	0.21	0.23	0.30	0.19	0.18	0.22	0.20
Social 17								—	0.21	0.25	0.28	0.24	0.19	0.26	0.30	0.22
Task 1									—	0.52	0.56	0.48	0.41	0.50	0.39	0.51
Task 3										—	0.55	0.49	0.39	0.47	0.46	0.49
Task 5											—	0.43	0.51	0.56	0.47	0.55
Task 8												—	0.44	0.55	0.50	0.47
Task 10													—	0.49	0.47	0.52
Task 14														—	0.45	0.53
Task 16															—	0.50
Task 18																—

**Table 3:** Goodness of fit summary for one- and two-factor models.

Model				IFI	TLI	NFI	CFI	RMSEA		
	*χ* ^2^	df	*χ* ^2^/df					Value	90% CI	*p* value
One-factor	543.356^*∗*^	170	3.102	0.78	0.68	0.75	0.81	0.084	0.075–0.093	<0.001
Two-factor	313.425^*∗*^	169	1.855	0.94	0.93	0.89	0.92	0.051	0.043–0.062	<0.001

^*∗*^ *p* value < 0.001.
