# Corrigendum to “Validity and Reliability of Farsi Version of Youth Sport Environment Questionnaire”

**DOI:** 10.1155/2015/149159

**Published:** 2015-10-12

**Authors:** Mohammad Ali Eshghi, Ramin Kordi, Amir Hossein Memari, Ahmad Ghaziasgar, Mohammad-Ali Mansournia, Seyed Hojjat Zamani Sani

**Affiliations:** ^1^Exercise Physiology Research Center, Baqiyatallah University of Medical Sciences, Tehran, Iran; ^2^Sports Medicine Research Center, Tehran University of Medical Sciences, P.O. Box 14395-578, Tehran, Iran; ^3^Department of Epidemiology and Biostatistics, School of Public Health, Tehran University of Medical Sciences, Tehran, Iran; ^4^Faculty of Physical Education and Sport Sciences, University of Tabriz, Tabriz, Iran

In the paper entitled “Validity and Reliability of Farsi Version of Youth Sport Environment Questionnaire,” [1] there was an error in the 4th institutional affiliation and it is corrected above.
