# Supplementary material for: Validity and Reliability of Farsi Version of Youth Sport Environment Questionnaire
Source: J Sports Med (Hindawi Publ Corp). 2015 Aug 12;2015:985283. doi: 10.1155/2015/985283 (PMC4590900; doi:10.1155/2015/985283)
Supplement: Supplementary file 1 — Supplementary Material: Example items from the Youth Sport Environment Questionnaire. [file 985283.f1.pdf]

Supplemental material: Example items from the Youth Sport Environment Questionnaire

Questions	Strongly disagree--------------------- Strongly agree								
1. We all share the same commitment to our team's goals.	1	2	3	4	5	6	7	8	9
2. I invite my teammates to do things with me.	1	2	3	4	5	6	7	8	9
3. As a team, we are all on the same page.	1	2	3	4	5	6	7	8	9
4. Some of my best friends are in this team.	1	2	3	4	5	6	7	8	9
5. I like the way we work together as a team.	1	2	3	4	5	6	7	8	9
6. I do not get along with the members of my team.	1	2	3	4	5	6	7	8	9
